# Withdrawal

**DOI:** 10.1002/fsn3.3088

**Published:** 2022-10-19

Withdrawal: “Effects of water‐deficit stress and putrescine on performances, photosynthetic gas exchange, and chlorophyll fluorescence parameters of *Salvia officinalis* in two cutting times,” by Maryam Mohammadi‐Cheraghabadi, Seyed Ali Mohammad Modarres‐Sanavy, Fatemeh Sefidkon, Ali Mokhtassi‐Bidgoli, and Saeid Hazrati, *Food Science & Nutrition*, 2022, 10, 1431–1441. https://doi.org/10.1002/fsn3.2741

The above article, published online in Volume 10, Issue 5 in Wiley Online Library (https://onlinelibrary.wiley.com/doi/full/10.1002/fsn3.2741), has been withdrawn by agreement between the Journal's Editor‐in‐Chief, Prof. Y. Martin Lo, and Wiley Periodicals, LLC. The withdrawal has been agreed because the article was published in error after having been rejected. The authors bear no fault, and we apologize for the error.

